# Effects of aspirin on risk and severity of early recurrent stroke after transient ischaemic attack and ischaemic stroke: time-course analysis of randomised trials

**DOI:** 10.1016/S0140-6736(16)30468-8

**Published:** 2016-07-23

**Authors:** Peter M Rothwell, Ale Algra, Zhengming Chen, Hans-Christoph Diener, Bo Norrving, Ziyah Mehta

**Affiliations:** a Stroke Prevention Research Unit, Nuffield Department of Clinical Neurosciences, University of Oxford, Oxford, UK; b Department of Neurology, Rudolph Magnus Institute for Neuroscience, and Julius Center for Health Sciences and Primary Care, University Medical Center Utrecht, Utrecht, Netherlands; c Nuffield Department of Population Health, University of Oxford, Oxford, UK; d Department of Neurology, University Duisburg-Essen, Essen, Germany; e Department of Clinical Sciences, Section of Neurology, Lund University, Sweden

## Abstract

**Background:**

Aspirin is recommended for secondary prevention after transient ischaemic attack (TIA) or ischaemic stroke on the basis of trials showing a 13% reduction in long-term risk of recurrent stroke. However, the risk of major stroke is very high for only the first few days after TIA and minor ischaemic stroke, and observational studies show substantially greater benefits of early medical treatment in the acute phase than do longer-term trials. We hypothesised that the short-term benefits of early aspirin have been underestimated.

**Methods:**

Pooling the individual patient data from all randomised trials of aspirin versus control in secondary prevention after TIA or ischaemic stroke, we studied the effects of aspirin on the risk and severity of recurrent stroke, stratified by the following time periods: less than 6 weeks, 6–12 weeks, and more than 12 weeks after randomisation. We compared the severity of early recurrent strokes between treatment groups with shift analysis of modified Rankin Scale (mRS) score. To understand possible mechanisms of action, we also studied the time course of the interaction between effects of aspirin and dipyridamole in secondary prevention of stroke. In a further analysis we pooled data from trials of aspirin versus control in which patients were randomised less than 48 h after major acute stroke, stratified by severity of baseline neurological deficit, to establish the very early time course of the effect of aspirin on risk of recurrent ischaemic stroke and how this differs by severity at baseline.

**Findings:**

We pooled data for 15 778 participants from 12 trials of aspirin versus control in secondary prevention. Aspirin reduced the 6 week risk of recurrent ischaemic stroke by about 60% (84 of 8452 participants in the aspirin group had an ischaemic stroke *vs* 175 of 7326; hazard ratio [HR] 0·42, 95% CI 0·32–0·55, p<0·0001) and disabling or fatal ischaemic stroke by about 70% (36 of 8452 *vs* 110 of 7326; 0·29, 0·20–0·42, p<0·0001), with greatest benefit noted in patients presenting with TIA or minor stroke (at 0–2 weeks, two of 6691 participants in the aspirin group with TIA or minor stroke had a disabling or fatal ischaemic stroke *vs* 23 of 5726 in the control group, HR 0·07, 95% CI 0·02–0·31, p=0·0004; at 0–6 weeks, 14 *vs* 60 participants, 0·19, 0·11–0·34, p<0·0001). The effect of aspirin on early recurrent ischaemic stroke was due partly to a substantial reduction in severity (mRS shift analysis odds ratio [OR] 0·42, 0·26–0·70, p=0·0007). These effects were independent of dose, patient characteristics, or aetiology of TIA or stroke. Some further reduction in risk of ischaemic stroke accrued for aspirin only versus control from 6–12 weeks, but there was no benefit after 12 weeks (stroke risk OR 0·97, 0·84–1·12, p=0·67; severity mRS shift OR 1·00, 0·77–1·29, p=0·97). By contrast, dipyridamole plus aspirin versus aspirin alone had no effect on risk or severity of recurrent ischaemic stroke within 12 weeks (OR 0·90, 95% CI 0·65–1·25, p=0·53; mRS shift OR 0·90, 0·37–1·72, p=0·99), but dipyridamole did reduce risk thereafter (0·76, 0·63–0·92, p=0·005), particularly of disabling or fatal ischaemic stroke (0·64, 0·49–0·84, p=0·0010). We pooled data for 40 531 participants from three trials of aspirin versus control in major acute stroke. The reduction in risk of recurrent ischaemic stroke at 14 days was most evident in patients with less severe baseline deficits, and was substantial by the second day after starting treatment (2–3 day HR 0·37, 95% CI 0·25–0·57, p<0·0001).

**Interpretation:**

Our findings confirm that medical treatment substantially reduces the risk of early recurrent stroke after TIA and minor stroke and identify aspirin as the key intervention. The considerable early benefit from aspirin warrants public education about self-administration after possible TIA. The previously unrecognised effect of aspirin on severity of early recurrent stroke, the diminishing benefit with longer-term use, and the contrasting time course of effects of dipyridamole have implications for understanding mechanisms of action.

**Funding:**

Wellcome Trust, the National Institute of Health Research (NIHR) Biomedical Research Centre, Oxford.

## Introduction

The risk of recurrent stroke is up to 10% in the week after a transient ischaemic attack (TIA) or minor stroke.^1^, ^2^, ^3^, ^4^ Urgent medical treatment seems to reduce that risk by as much as 80%,^5^, ^6^ but many patients delay seeking medical attention, often for several days or weeks, even when they make a correct self-diagnosis.^7^, ^8^ Public education campaigns, such as the FAST test television campaign, have decreased delays to presentation after major stroke,^9^, ^10^ but there has been little improvement in presentation rates after TIA or minor stroke (appendix p 1).^11^ In a recent population-based study in the UK, half of recurrent strokes in the days after a TIA occurred prior to medical attention being sought for the initial event,^11^ and the situation is likely to be worse in many parts of the developing world in which access to emergency services is poor.

**Research in context**

**Evidence before this study**

Previous systematic reviews of randomised trials of aspirin versus placebo in secondary prevention after transient ischaemic attack (TIA) or ischaemic stroke reported only a 13% relative reduction in risk of recurrent stroke. Systematic reviews of trials of aspirin in treatment of hospitalised patients with acute stroke also reported a 13% reduction in the short-term risk of recurrent stroke or intracerebral haemorrhage. Yet, observational studies have suggested much more substantial benefits of urgent medical treatment after TIA or minor stroke, with the early risk of recurrent stroke reduced by as much as 80%, and a possible reduction in severity of stroke. The time course of benefit of aspirin had not been studied in randomised trials or in any subsequent systematic reviews. Therefore, we did a pooled analysis of individual patient data from all available trials of aspirin versus control after TIA or ischaemic stroke.

**Added value of this study**

Our analyses of data from trials of aspirin in secondary prevention after TIA and ischaemic stroke show that the effect of aspirin on risk of early recurrent events has been underestimated. We show substantial reductions in the early risk of all stroke, ischaemic stroke, and acute myocardial infarction. We also found that a major part of the early benefit of aspirin was due to a previously unrecognised reduction in severity of early recurrent ischaemic stroke, resulting in 80–90% reductions in the early risk of disabling or fatal recurrent ischaemic stroke after TIA and minor stroke. Although these trials recruited few patients in first few days after TIA or stroke, we found similar reductions in risk of recurrent ischaemic stroke with aspirin in trials of acute ischaemic stroke.

**Interpretation of all available evidence**

Urgent medical treatment substantially reduces the risk of early recurrent stroke after TIA and minor stroke and early use of aspirin is the key intervention. Medical services should give aspirin as soon as possible and public education should be aimed at self-administration after unfamiliar transient neurological symptoms suggestive of threatened stroke.

Antithrombotic treatment is important in the immediate management of most acute ischaemic vascular events.^12^, ^13^ Since aspirin is available in many households, public education materials recommend self-administration by patients who develop acute chest pain, in addition to seeking immediate medical attention.^14^, ^15^ Prehospital self-administration of aspirin is discouraged after stroke^15^ because of concerns about possible intracerebral haemorrhage. However, haemorrhage is a rare cause of TIA symptoms and it accounts for less than 5% of minor strokes.^16^, ^17^ Although public education should continue to persuade people with transient neurological symptoms to seek medical attention immediately, where this is possible, self-administration of aspirin after transient unfamiliar symptoms might also be appropriate, particularly in rural settings or in less developed countries where access to medical services will be delayed.

There are, however, few published data from randomised trials for the effect of aspirin on risk of early recurrent stroke after TIA and minor stroke, and no data for its effect on severity; evidence of apparently major benefits of urgent medical treatment generally comes only from observational studies.^5^, ^18^ Randomised trials of aspirin versus placebo in longer-term secondary prevention showed only a 13% relative reduction in risk of recurrent stroke with aspirin.^12^, ^13^, ^19^ Trials of short-term treatment of hospitalised acute stroke also reported a 13% reduction in the 4 week risk of recurrent stroke or intracerebral haemorrhage, but the effect of aspirin on risk or severity of recurrence after more minor stroke was not reported.^20^, ^21^, ^22^ Yet, observational studies suggest potentially substantial early benefits of aspirin after TIA or minor stroke. In the EXPRESS study, urgent treatment with antiplatelet drugs, blood pressure-lowering drugs, and statins reduced the early risk of stroke by 80%;^5^, ^6^ much of this decrease was hypothesised to have been due to aspirin.^5^ Severity of recurrent cerebral events was also reduced in EXPRESS (appendix p 2), which might also have been due to aspirin.

In the absence of published randomised evidence of the effect of aspirin on risk and severity of early recurrent stroke after TIA and minor stroke, we reanalysed individual patient data and reviewed original paper records on early outcomes from all available trials of aspirin versus placebo in secondary prevention after TIA or ischaemic stroke. To inform on possible mechanisms of action, we also aimed to study the time course of the interaction between effects of aspirin and dipyridamole in secondary prevention of stroke. Aiming to more reliably estimate the very early time course of onset of effects of aspirin, we also studied risk of recurrent ischaemic stroke in trials of aspirin in treatment of acute stroke, stratified by severity of the pre-randomisation neurological deficit.

## Methods

### Data selection and extraction

Trials were eligible if they randomised the following: patients with TIA or ischaemic stroke to regular aspirin (any dose; in the presence or absence of another antiplatelet drug) versus no antiplatelet or anticoagulant in the secondary prevention of stroke and other vascular events; patients with acute ischaemic stroke to regular aspirin (any dose) versus no aspirin, in the presence or absence of another antithrombotic treatment, for acute treatment and prevention of early recurrence; or patients with TIA or ischaemic stroke to regular dipyridamole (any dose) versus no dipyridamole (in the presence or absence of another antiplatelet drug) in the secondary prevention of stroke and other vascular events. With searches done up to Jan 31, 2016, PMR identified trials through searches of the Antithrombotic Trialists' (ATT) Collaboration,^13^, ^23^ subsequent systematic reviews, and the Cochrane Collaboration.^24^, ^25^ In view of the historical nature of the trials, no additional searches were made for ongoing trials or abstracts presented at meetings.

For all eligible trials of aspirin or dipyridamole in secondary prevention after TIA or stroke, we sought to obtain individual patient data. If these were not available, published data on vascular events were extracted from trial reports. Data were obtained on the following baseline variables: randomised treatment allocation, age, sex, prior diabetes, current smoking, history of hypertension, blood pressure at entry, time from most recent cerebrovascular event to randomisation, nature of the most recent cerebrovascular event prior to randomisation (TIA; “minor” or non-disabling stroke; “major” or disabling stroke), and premorbid disability (modified Rankin Scale [mRS] score). Data were also obtained on the nature and timing of the following outcome variables: any recurrent stroke, recurrent ischaemic stroke, acute myocardial infarction, intracerebral haemorrhage, fatal extracranial bleeding, and cause of any other deaths. If available, from either electronic or paper records, data were obtained on the severity and outcome of all recurrent strokes (mRS score). There were minor differences in definition of recurrent stroke between trials, but designations made in the original trials were not changed.

For trials of aspirin in treatment of major acute stroke, we also sought to obtain individual patient data on the severity of stroke at entry (eg, a severity score or other measure of the extent on the baseline neurological deficit) and on time to first recurrent ischaemic stroke during the trial period. In one small trial,^26^ only data for progression of stroke were collected (defined as a worsening of at least 2 points on the Scandanavian Stroke Progression Scale^27^). In the absence of any other data, this outcome was included for completeness.

### Statistical analysis

All analyses were by intention to treat based on the randomised treatment allocation. In the secondary prevention trials, we calculated the effects of aspirin versus control and dipyridamole versus control, with stratification by time from randomisation (0–6 weeks, 6–12 weeks, and >12 weeks), for the following outcomes: recurrent ischaemic stroke, disabling or fatal recurrent ischaemic stroke, any recurrent stroke, any fatal stroke, intracerebral haemorrhage, and acute myocardial infarction. For each outcome, we calculated odds ratios (OR) for each trial and pooled estimates by fixed-effects meta-analysis (Mantel-Haenszel-Peto method) if there was no significant heterogeneity (χ^2^ test) between trials. In the absence of significant heterogeneity, we pooled individual patient data and generated Kaplan-Meier curves (1–proportion free of event) for time to first event. Statistical significance of any effect of randomised treatment allocation was determined using the log-rank test stratified by trial and hazard ratios (HRs) and 95% CIs were generated for events up to 12 weeks follow-up using Cox proportional hazards models stratified by trial (the assumption of proportional hazards was violated if events after 12 weeks were included). We compared the severity of early recurrent strokes between treatment groups based on mRS scores with ordinal regression (mRS shift) analysis. The assumption of proportional odds was assessed with the score test and was valid (p>0·3) for all analyses. However, we also calculated ORs for the traditional single cutoff point of an mRS score of higher than 2. Stratified analyses of the preventive effect of treatment on recurrent ischaemic stroke and on disabling or fatal recurrent ischaemic stroke were also done for the following potential effect modifiers: dose of aspirin (<100 mg [low] *vs* ≥300 mg [high]), TIA or minor stroke only versus major stroke (usually defined as the presence of residual neurological signs) at baseline; time from last TIA or minor stroke to randomisation (≤14 days *vs* >14 days), age (<65 years *vs* 65–74 years *vs* ≥75 years), sex, diabetes, current smoking, and hypertension (prior diagnosis or blood pressure ≥160/90 mm Hg at baseline assessment *vs* none).

In trials of aspirin for acute stroke, we determined the effect of aspirin versus control on risk of recurrent ischaemic stroke. Trials differed in duration of randomised treatment allocation (appendix p 3). To maximise comparativeness between the trials, we first determined the effect of aspirin up to day 14 after randomisation, stratified by the severity of the stroke at the baseline assessment. In the two largest trials, data for the extent of baseline neurological deficit had been collected in the same way, with documentation of the presence or absence of neurological deficits of the following types: face, arm or hand, leg or foot, dysphasia, visuospatial, brainstem or cerebellar, hemianopia, and other. We quantified the severity of stroke as follows: mild (≤2 deficits), moderately severe (3–4 deficits), and severe (≥5 deficits). A third smaller trial had quantified baseline severity of stroke using approximate quartile categories of the Scandinavian Stroke Progression Scale^27^ score (<9, 10–11, 12–13, and 14–25). The distribution of severity that most closely matched that in the other two trials was: mild (<9), moderately severe (10–14), or severe (14–25). We calculated ORs for the effect of aspirin on the 14 day risk of recurrent ischaemic stroke for each trial, with stratification by severity of initial stroke. Pooled estimates were obtained by fixed-effects meta-analysis (Mantel-Haenszel-Peto method) if there was no significant heterogeneity (p>0·05 on χ^2^ test) between trials, or otherwise by random-effects meta-analysis. To determine the time course of onset of effect of aspirin in the acute phase, we did a pooled analysis, stratified by time from randomisation to recurrent ischaemic stroke (days 0–1, 2–3, 4–6, 7–14, and ≥15). This analysis covered the full period of randomised treatment allocation in the trials and was done both with and without the patients in the International Stroke Trial (IST)^20^ who had been taking aspirin prior to randomisation. Prior aspirin use was an exclusion criterion in the smaller trial^26^ and was rare in the Chinese Acute Stroke Trial (CAST).^21^

### Role of the funding source

The funders of the study had no role in study design, data collection, data analysis, data interpretation, or writing of the report. The corresponding author had full access to all the data in the study and had final responsibility for the decision to submit for publication.

## Results

We identified 12 trials of 15 778 participants that assessed aspirin versus control in secondary prevention after TIA or ischaemic stroke (appendix p 3). Data for recurrent vascular events within 12 weeks of randomisation were available for all these trials. 11 trials included comparisons of aspirin alone versus placebo, and three trials included comparisons of aspirin plus dipyridamole versus no aspirin.

**Figure 1:**
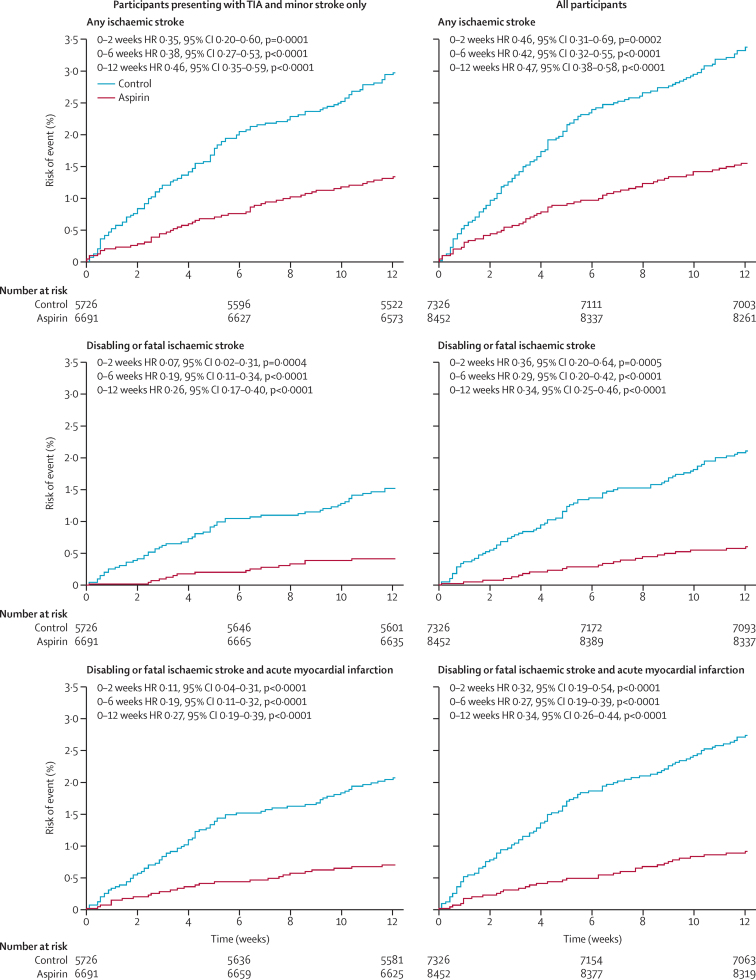
Pooled analysis of the early risk of recurrent vascular events in 12 trials of any aspirin versus control Statistical significance calculated with the log-rank test. TIA=transient ischaemic attack. HR=hazard ratio.

**Figure 2:**
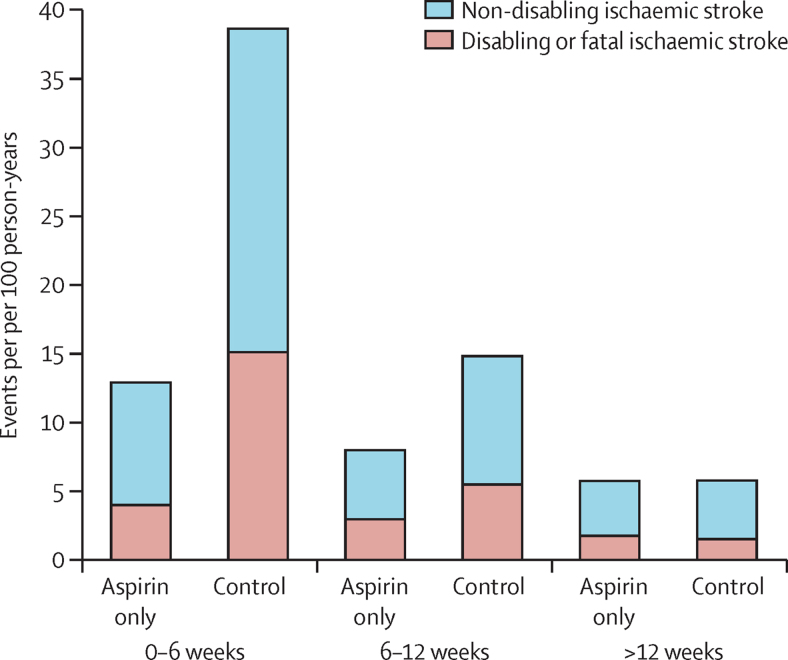
Pooled analysis of the effect of aspirin only versus control in secondary prevention after transient ischaemic attack and ischaemic stroke on the absolute risk of recurrent ischaemic stroke Time course of treatment effect interaction: p_interaction_<0·0001 for both outcomes.

Among 9635 participants in the 11 trials of aspirin only versus control, aspirin reduced the 6 week risk of recurrent ischaemic stroke by about 60% (HR 0·41, 95% CI 0·30–0·56; p<0·0001; table 1), with a similar effect at 12 weeks (table 1) and no heterogeneity between trials (p_het_=0·85; appendix p 5). Inclusion of data from the three additional trials for comparisons for aspirin plus dipyridamole versus no aspirin (ie, any aspirin) did not change the result (HR 0·42, 95% CI 0·32–0·55, p<0·0001; table 1). Benefit was greatest at 0–2 weeks (figure 1), but further benefit accrued up to 12 weeks follow-up (table 1, Figure 1, Figure 2).

**Figure 3:**
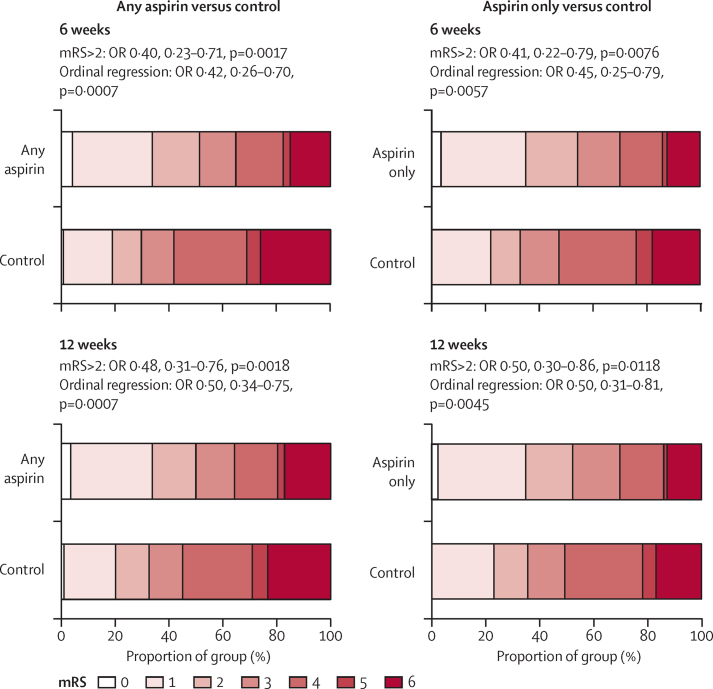
Pooled analysis of the effect of aspirin versus control on the severity (mRS score on follow-up) of recurrent ischaemic stroke in the first 6 weeks and the first 12 weeks after randomisation in trials in secondary prevention after transient ischaemic attack and ischaemic stroke OR=odds ratio. mRS=modified Rankin Scale.

Aspirin also reduced the severity of recurrent ischaemic stroke during the 6 weeks after randomisation (figure 3), with a similar effect seen at 12 weeks and when analyses were based only on an mRS score higher than 2 (figure 3). Consequently, aspirin reduced the 6 week risk of disabling or fatal (mRS score >2) ischaemic stroke by about 70% (table 1; p_het_=0·91) and the risk of very severe (mRs score 4–6) ischaemic stroke by about 75% (26 of 8452 participants in active group had an event *vs* 92 of 7326 in control group; HR 0·25, 0·16–0·39, p<0·0001), but had less effect on non-disabling (mRs ≤2) stroke (48 of 8452 participants had an event *vs* 65 of 7326; HR 0·64, 0·44–0·93, p=0·020). Benefit continued to accrue for risk of disabling or fatal ischaemic stroke up to 12 weeks follow-up (table 1, Figure 1, Figure 2), but the greatest reduction was seen within the first 2 weeks, particularly in patients presenting with TIA and minor stroke (two of 6691 participants in the aspirin group with TIA or minor stroke had a disabling or fatal ischaemic stroke *vs* 23 of 5726 in the control group, 95% CI HR 0·07, 0·02–0·31, p=0·0004; figure 1).

Aspirin also reduced the early risks of any recurrent stroke, fatal stroke, and acute myocardial infarction (table 1). There was no increase in the 12 week risk of intracerebral haemorrhage on low-dose aspirin versus control (three of 4125 participants in active group had an event *vs* five of 4137 in the control group), but there was a trend towards higher risk for high-dose aspirin compared with control (five of 4297 *vs* one of 3159; HR 3·68, 95% CI 0·43–31·51, p=0·23). However, four of the events on high-dose aspirin occurred as complications of carotid endarterectomy in the UK-TIA Aspirin Trial (three in patients assigned to 1200 mg of aspirin and one to 300 mg). There was only one fatal extracranial bleed within 12 weeks of randomisation in any of the trials (on aspirin plus dipyridamole in the European Stroke Prevention Study [ESPS]-2).

The effect of aspirin on the 12 week risk of recurrent ischaemic stroke was independent of dose and patient characteristics (table 2, figure 1, appendix p 6). In trials where data were available (5606 assigned to aspirin *vs* 4803 to control), we found a similar effect on 12 week risk of disabling or fatal recurrent ischaemic stroke in patients with atrial fibrillation at baseline (HR 0·28, 95% CI 0·08–1·00, p=0·0508) and in those with lacunar stroke (data in ESPS-2 only; data not shown). There was also no interaction between time from the last TIA or stroke to randomisation and the effect of any aspirin versus control on the 12 week risks of ischaemic stroke or of disabling or fatal ischaemic stroke (table 2). Aspirin reduced the 12 week risk of disabling or fatal ischaemic stroke in patients recruited fewer than 14 days after their last event (HR 0·46, 0·27–0·77, p=0·0035), but there were too few patients recruited within 7 days to define the effect of treatment during this time.

The absolute risk of recurrent ischaemic stroke fell with time from randomisation (p_interaction_<0·0001; figure 2). In trials of aspirin only versus control, there was no reduction in risk of recurrent ischaemic stroke after 12 weeks (OR 0·97, 95% CI 0·84–1·12, p=0·67; p_int_<0·0001 for <12 weeks *vs* >12 weeks), with no heterogeneity between trials (appendix p 7). There was also no reduction in severity of post-12-week strokes (mRS shift OR 1·00, 95% CI 0·77–1·29, p=0·97).

We identified eight trials (11 937 participants) of dipyridamole versus control (with or without aspirin) in secondary prevention after TIA or ischaemic stroke (appendix p 3). Three trials with comparisons of aspirin plus dipyidamole versus no antiplatelet were included in the any aspirin versus control analyses described above. Seven trials (9437 participants) included comparisons of dipyridamole plus aspirin versus aspirin alone, and one trial (6602 participants) also included comparisons of dipyridamole versus aspirin and dipyridamole versus control (appendix p 3). Data for recurrent vascular events within 12 weeks of randomisation were obtained for all of these trials. Adding dipyridamole to aspirin had no effect on the 12 week risk of recurrent ischaemic stroke (OR 0·90, 95% CI 0·65–1·25, p=0·53; appendix p 11), with no heterogeneity between trials (p_het_=0·31), and had no effect on severity of recurrent ischaemic stroke within 12 weeks of randomisation (mRS shift analysis OR 0·90, 95% CI 0·37–1·72, p=0·99). However, adding dipyridamole to aspirin did reduce the risk of recurrent ischaemic stroke after 12 weeks (OR 0·76, 95% CI 0·63–0·92, p=0·005), particularly the risks of disabling or fatal ischaemic stroke (0·64, 0·49–0·84, p=0·0010) and any disabling or fatal stroke (0·65, 0·51–0·84, p=0·0008). Dipyridamole versus control also had no effect on severity of 12 week recurrent ischaemic stroke in the ESPS-2 trial (mRS shift analysis for any dipyridamole *vs* control OR 0·98, 95% CI 0·58–1·66, p=0·95; dipyridamole only *vs* control OR 1·11, 0·59–2·09, p=0·74).

**Figure 4:**
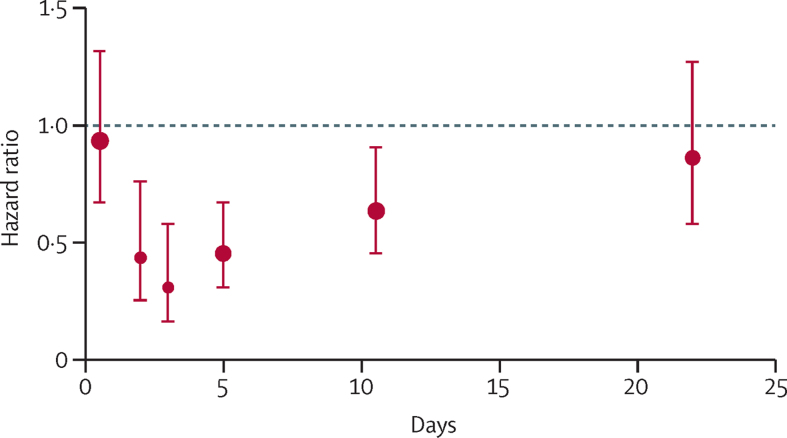
Pooled hazard ratios for the effect of aspirin versus control on risk of recurrent ischaemic stroke in patients with mild and moderately severe initial neurological deficits during early follow-up in Chinese Acute Stroke Trial and International Stroke Trial^20^, ^21^ Data plotted at median timepoint for the following follow-up periods from randomisation: days 0–1, days 2–3, days 4–6, days 7–14, after 15 days. Error bars show 95% CIs. This analysis excludes 3292 (21%) patients with mild or moderately severe stroke in the International Stroke Trial who had received aspirin during the days before randomisation. The equivalent analysis with these patients included is in appendix p 6.

Given the small numbers of patients randomised within 7 days of their last event in the trials of aspirin in secondary prevention after TIA or stroke, we studied the time course of risk of recurrent ischaemic stroke in trials of aspirin in treatment of acute stroke, in which all patients were randomised within 48 h of stroke onset. Among three eligible trials (40 531 participants, appendix p 8), individual patient data were available from the two largest (40 090 participants) and tabular data from the smaller trial (441 participants). The effect of aspirin versus control on the 14 day risk of recurrent ischaemic stroke differed in relation to the severity of stroke (p_het_=0·014), as indicated by the extent of the baseline neurological deficit (appendix p 9). Aspirin reduced the 14 day risk of recurrent ischaemic stroke in participants with mild neurological deficits at baseline, with a consistent effect across the trials, but had no effect in those with severe deficits at baseline (appendix p 9). In participants with moderately severe deficits, there was a significant overall reduction in risk, but there was heterogeneity between trials (appendix p 9). There was no interaction (p=0·92) between the effect of aspirin on recurrent ischaemic stroke and randomisation between heparin and no heparin in the IST (data not shown). On pooled analysis of recurrent ischaemic stroke in patients with mild and moderately severe initial deficits, no effect of aspirin was found within the first 24 h (figure 4) after randomisation, but risk was reduced by day 2 (HR 0·44, 95% CI 0·25–0·76, p=0·0034) and day 3 (0·31, 0·16–0·58, p=0·0003), with further reductions during days 4–6 (0·45, 0·31–0·67, p<0·0001) and days 7–14 (0·64, 0·45–0·91, p=0·0121), but not after 14 days (0·86, 0·58–1·27, p=0·45; figure 4). Results were similar after inclusion of 3292 (21%) participants in IST who had received aspirin during the days before randomisation (figure 4, appendix p 10). Of note, allocation to continued aspirin in this group did reduce the risk of recurrent ischaemic stroke in the first 24 h (HR 0·31, 95% CI 0·11–0·85, p=0·020).

## Discussion

Our analyses of trials of aspirin in secondary prevention after TIA and ischaemic stroke show that the effect of aspirin on early recurrent events has been underestimated. We identified substantial reductions in the early risks of all stroke, ischaemic stroke, and acute myocardial infarction with aspirin, with effect sizes greater than those previously reported after unstable angina or acute myocardial infarction.^23^ We also found that a major part of the early benefit of aspirin was due to an hitherto unrecognised reduction in severity of early recurrent ischaemic stroke, resulting in up to a 90% reduction in early risk of disabling or fatal recurrent ischaemic stroke after TIA and minor stroke.

However, trials of aspirin for secondary prevention recruited few patients in the acute phase after TIA or stroke. We found no significant diminution of the effect in patients randomised early, but acute effects might differ. For example, aspirin had no effect on death for the first 3 days after acute myocardial infarction in the ISIS-2 trial.^28^ We therefore studied trials of aspirin in treatment of acute stroke, aiming simply to estimate the time course of onset of effect of aspirin on risk of recurrent ischaemic stroke, with the overall balance of risk and benefit having been documented elsewhere.^20^, ^21^, ^22^ With use of stratification by severity of the baseline neurological deficit, we showed that in patients with less severe stroke the relative reduction in risk of recurrent ischaemic stroke on aspirin was similar to that in the secondary prevention trials and was evident by the second full day of treatment. Aspirin could also have reduced further thrombosis, or related processes, in patients with more major stroke, but such effects would probably be less clinically evident in the territory of an already large cerebral infarct.

Our results have implications for acute treatment after TIA and minor stroke. First, they confirm findings from previous non-randomised studies for the impact of urgent treatment on the early risk of recurrent stroke,^5^, ^6^, ^18^ supporting recommendations for urgent assessment of patients. Second, they suggest that most of the benefit of urgent treatment in these previous multi-intervention studies was simply due to aspirin. Therefore, it is essential that patients with TIA or minor stroke are not sent home from the emergency department with advice to add aspirin to their next prescription; they should be treated acutely. Similarly, patients who telephone their family doctor or advice lines should be told to take aspirin immediately if TIA is suspected, in addition to obtaining medical attention. Aspirin could also be given by paramedics when they assess patients at home. Third, we showed that aspirin reduced early recurrent stroke in non-anticoagulated patients with atrial fibrillation at baseline.

Our findings also have implications for the choice of antithrombotic treatment early after TIA or ischaemic stroke. Most guidelines do not distinguish between the early and later phases of secondary prevention and several recommend clopidogrel monotherapy or other drugs as equal alternatives to aspirin.^29^, ^30^ Our findings suggest that treatment in the first few days and weeks should include aspirin unless some other antithrombotic agent is shown to be superior. We found that dipyridamole monotherapy was inferior to aspirin in prevention of early recurrent stroke, and that addition of dipyridamole to aspirin did not enhance the effects of aspirin on risk or severity of early recurrent ischaemic stroke. Clopidogrel plus aspirin does appear to be more effective than aspirin alone in prevention of early recurrent stroke after TIA and minor ischaemic stroke,^31^, ^32^ but has no effect on severity of stroke,^33^ and the only trial of clopidogrel monotherapy versus aspirin plus dipyridamole in this patient group reported data that, in light of our findings, suggest increased severity of early recurrent stroke in the clopidogrel group.^34^ Indeed, although the PROFESS trial showed no difference in overall severity of recurrent stroke on aspirin plus dipyridamole versus clopidogrel in secondary prevention after TIA and ischaemic stroke,^35^ our findings suggest that risk and severity of early recurrent stroke might have been reduced by aspirin plus dipyridamole in patients who were randomised soon after their initial TIA or stroke, such that the early effects of aspirin would not already be lost after prolonged pre-randomisation use. Diminishing benefit of aspirin with longer pre-randomisation use would also explain why the advantage of aspirin plus clopidogrel compared with clopidogrel alone in the MATCH trial was only seen in patients randomised early after their TIA or stroke,^36^ and possibly why previous observational studies of severity of ischaemic stroke in relation to previous aspirin use have yielded conflicting results. Similar considerations will apply to trials of cilostazol, ticagrelor, and future novel anticoagulants in secondary prevention of stroke. In fact, survival curves in trials of aspirin versus cilostazol suggest that aspirin is superior for the first 3 months, but cilostazol is more effective thereafter.^37^, ^38^ Future trials of new antiplatelet or antithrombotic drugs in prevention of early recurrent stroke should report data for severity of stroke and should avoid mixing patients taking aspirin with those taking other antiplatelet drugs in the comparator arm.

Our findings also have implications for public education. First, confirmation that urgent treatment after TIA and minor ischaemic stroke reduces the early risk of disabling or fatal stroke by about 80% highlights the need to reduce delays in patients seeking medical attention. Second, since aspirin is available in many households, consideration should be given to promoting self-administration immediately after transient stroke-like neurological symptoms, as is recommended for people who have acute chest pain.^14^, ^15^ Intracranial haemorrhage is rare in patients with TIA symptoms and accounts for less than 5% of minor strokes.^16^, ^17^ Moreover, randomised trials of antithrombotic drugs that have included patients with acute intracranial haemorrhage have not shown any increase in risk of death or of recurrent haemorrhage.^39^ Similarly, there is no evidence that prior aspirin would worsen outcome in the small proportion of patients who still subsequently progressed to have a major stroke and required thrombolysis or thrombectomy.^40^, ^41^ Indeed, given the substantial reductions in progression to disabling or fatal early recurrent ischaemic stroke that we noted with aspirin, prevention of the need for thrombolysis or thrombectomy will be the main benefit. Public education should continue to persuade people with transient unfamiliar neurological symptoms to seek medical attention immediately, where this is possible, but self-administration of aspirin would also be prudent, particularly in rural settings or in less developed countries where access to emergency services might be delayed. Some individuals would take an aspirin or two unnecessarily, as is the case with non-cardiac chest pain, but others would benefit.

For longer-term secondary prevention after TIA and ischaemic stroke, aspirin had no significant effect on risk or severity of recurrent ischaemic stroke after 12 weeks. However, early benefit of aspirin was maintained on longer-term follow-up, even though no additional benefit accrued. More detailed analyses of trials in a broader range of secondary prevention settings, and including all relevant outcomes, are underway to establish the longer-term balance of risk and benefit (Rothwell PM, unpublished), and new trials should determine the risk and benefits of stopping aspirin. The absence of additional benefit after 12 weeks does, however, necessitate re-interpretation of trials of long-term secondary prevention of stroke with other antithrombotic drugs versus aspirin that have shown either no benefit of the other drug^42^ or only a small benefit.^43^ The interpretation that these drugs are as effective as aspirin is less positive if aspirin is ineffective.

We found no evidence that adding dipyridamole to aspirin reduced the risk or severity of early recurrent ischaemic stroke. However, dipyridamole plus aspirin versus aspirin alone did reduce the risk of later recurrent ischaemic stroke and this effect was particularly marked for disabling or fatal events. Further work is required to fully understand the nature of this late effect in the trials that we studied here and in the PROFESS trial.^35^

Our results do have limitations. First, the trials of aspirin in secondary prevention were done in the 1980s and 1990s. Medical care has since changed, with more detailed investigations and more intensive lowering of blood pressure and lipids. However, the effect of urgent treatment after TIA and minor stroke that was observed in more recent observational studies^5^, ^6^, ^18^ is very similar to that in the previous trials (appendix p 2). Changes in medical care would also impact less on the effectiveness of prehospital self-administration. Second, most patients in the secondary prevention trials were already beyond the very early high risk period after their initial TIA or minor stroke when recruited.^44^ However, delayed inclusion is likely to have underestimated the absolute reduction in risk of early recurrent stroke, as might the absence of a loading dose in the trials of low-dose aspirin, but relative risk reductions are likely to be generalisable to the acute phase. The absolute reduction in risk of ischaemic stroke in the EXPRESS study in which patients were treated more acutely was about 8% (number needed to treat 12; appendix p 2).^5^ We did not find a reduction in recurrent ischaemic stroke during the first 24 h in trials of aspirin in the acute treatment of major stroke (figure 4), but early deterioration after major stroke is multifactorial and recurrent stroke is difficult to distinguish from progression of existing pathological processes. There was no evidence of a delay in onset of benefit of acute treatment after TIA and minor stroke in observational studies in the acute phase.^5^, ^6^, ^18^ Third, some patients in the trials of aspirin in secondary prevention were treated with aspirin or other antithrombotic drugs for a short time after their initial TIA or stroke, prior to inclusion. However, we found no evidence that such treatment influenced the effect of subsequent randomised treatment, either in the SALT trial (appendix p 5), which had a short on-treatment run-in, or in the other secondary prevention trials (data not shown). However, prior aspirin use was associated with benefit within the first 24 h in the IST trial (appendix p 10). Finally, we did not report data on whether non-compliance with trial treatment might explain the diminishing longer-term benefit of aspirin. However, preliminary analyses show little evidence of this (Rothwell PM, unpublished) and compliance was clearly sufficient to show the late benefits of dipyridamole plus aspirin versus aspirin alone.

Our findings raise questions about the mechanisms by which aspirin reduces the risk and severity of early recurrent ischaemic stroke and by which effectiveness diminishes with longer-term use. It is unusual for preventive treatments to reduce the risk of disabling stroke more than non-disabling stroke, which might suggest a neuroprotective effect of aspirin, possibly due to prostaglandin-mediated effects on the microvasculature.^45^, ^46^ However, the similarly large reduction in the early risk of myocardial infarction suggests reversal of short-term systemic platelet activation. The loss of benefit of aspirin in longer-term use is at odds with the maintenance of platelet COX-1 inhibition,^47^ although time-course data on bleeding time are less clear-cut.^48^ It is possible that aspirin is only clinically effective during periods when platelets are activated, or that platelets adapt to aspirin via upregulation of non-COX-mediated pathways, or that gradual acetylation of other proteins affects other important pathways.^49^

In conclusion, we show the validity of previous non-randomised studies that reported the considerable impact that medical treatment has on the early risk of recurrent stroke after TIA and minor ischaemic stroke and we have identified aspirin as the key component. It is essential that aspirin is given to patients with suspected TIA or minor stroke immediately. Indeed, a case can be made for public education about self-administration after transient unfamiliar neurological symptoms. The previously unrecognised reduction in severity of early recurrent ischaemic stroke by aspirin has important implications for clinical guidelines, interpretation of previous and future trials, and for understanding mechanism of action. More generally, our findings highlight the fact that to understand the effects of newer drugs in comparison to, or in combination with, older drugs, it is first necessary to fully understand the effects of the older drug.

## Figures and Tables

**Table 1:** Pooled analysis of the early risk of recurrent vascular events, given per time period after randomisation, in trials of aspirin versus control in secondary prevention after transient ischaemic attack and ischaemic stroke

	**0–6 weeks**				**6–12 weeks**				**0–12 weeks**			
	Events (n/N)		HR (95% CI)	p	Events (n/N)		HR (95% CI)	p	Events (n/N)		HR (95% CI)	p
	In active group	In control group			In active group	In control group			In active group	In control group		
**Any aspirin *vs* control**												
Any ischaemic stroke	84/8452	175/7326	0·42 (0·32–0·55)	<0·0001	48/8334	72/7105	0·60 (0·41–0·86)	0·0060	132/8452	247/7326	0·47 (0·38–0·58)	<0·0001
Disabling or fatal ischaemic stroke	36/8452	110/7326	0·29 (0·20–0·42)	<0·0001	23/8388	41/7170	0·48 (0·29–0·81)	0·0055	59/8452	151/7326	0·34 (0·25–0·46)	<0·0001
Any stroke	91/8452	178/7326	0·45 (0·35–0·58)	<0·0001	49/8327	75/7097	0·58 (0·41–0·84)	0·0036	140/8452	253/7326	0·49 (0·40–0·60)	<0·0001
Any fatal stroke	16/8452	42/7326	0·36 (0·20–0·63)	0·0005	10/8434	12/7278	0·71 (0·30–1·65)	0·43	26/8452	54/7326	0·44 (0·27–0·70)	0·0006
Acute myocardial infarction	6/8452	26/7326	0·21 (0·09–0·51)	0·0006	11/8387	25/7215	0·39 (0·19–0·81)	0·011	17/8452	51/7326	0·30 (0·17–0·52)	<0·0001
**Aspirin only *vs* control**												
Any ischaemic stroke	57/5213	118/4422	0·41 (0·30–0·56)	<0·0001	29/5133	42/4272	0·60 (0·37–0·96)	0·034	86/5213	160/4422	0·46 (0·35–0·60)	<0·0001
Disabling or fatal ischaemic stroke	26/5213	78/4422	0·29 (0·19–0·46)	<0·0001	15/5169	25/4314	0·48 (0·25–0·93)	0·028	41/5213	103/4422	0·34 (0·24–0·49)	<0·0001
Any stroke	62/5213	121/4422	0·43 (0·32–0·59)	<0·0001	30/5132	44/4271	0·59 (0·37–0·94)	0·026	92/5213	165/4422	0·47 (0·37–0·61)	<0·0001
Any fatal stroke	11/5213	22/4422	0·46 (0·22–0·95)	0·035	5/5200	7/4396	0·53 (0·17–1·69)	0·28	16/5213	29/4422	0·48 (0·26–0·88)	0·018
Acute myocardial infarction	5/5213	20/4422	0·23 (0·09–0·63)	0·0038	7/5175	18/4353	0·35 (0·15–0·85)	0·020	12/5213	38/4422	0·29 (0·15–0·56)	0·0002

Analysis of any aspirin versus control includes comparisons of aspirin plus dipyridamole versus control.

**Table 2:** Pooled analysis of the effect of any aspirin versus control in secondary prevention after TIA and ischaemic stroke on the early risk of any recurrent ischaemic stroke and on disabling or fatal ischaemic stroke stratified by the nature of the presenting event (TIA and minor stroke *vs* major stroke) and by time from presenting event to randomisation (≤14 days *vs* >14 days)

		**0–6 weeks**			**6–12 weeks**			**0–12 weeks**			**p**_**interaction**_
		Events	HR (95% CI)	p	Events	HR (95% CI)	p	Events	HR (95% CI)	p	
**Presenting event**											
Any ischaemic stroke											
	TIA or minor stroke	169	0·38 (0·27–0·53)	<0·0001	92	0·63 (0·41–0·95)	0·028	261	0·46 (0·35–0·59)	<0·0001	0·75
	Major stroke	90	0·50 (0·32–0·77)	0·0018	28	0·51 (0·23–1·10)	0·088	118	0·50 (0·34–0·73)	0·0004	..
Disabling or fatal ischaemic stroke											
	TIA or minor stroke	74	0·19 (0·11–0·34)	<0·0001	41	0·43 (0·22–0·82)	0·010	115	0·26 (0·17–0·40)	<0·0001	0·15
	Major stroke	72	0·42 (0·25–0·69)	0·0007	23	0·59 (0·25–1·36)	0·21	95	0·46 (0·30–0·70)	0·0004	..
**Time since last event**											
Any ischaemic stroke											
	Time since last event ≤14 days	80	0·54 (0·34–0·85)	0·0082	34	0·66 (0·33–1·31)	0·24	114	0·57 (0·39–0·84)	0·0042	0·15
	Time since last event >14 days	131	0·34 (0·23–0·50)	<0·0001	72	0·56 (0·34–0·90)	0·016	203	0·41 (0·30–0·55)	<0·0001	..
Disabling or fatal ischaemic stroke											
	Time since last event ≤14 days	43	0·41 (0·21–0·78)	0·0071	21	0·58 (0·24–1·40)	0·22	64	0·46 (0·27–0·77)	0·0035	0·13
	Time since last event >14 days	77	0·21 (0·12–0·38)	<0·0001	36	0·45 (0·22–0·90)	0·025	113	0·28 (0·18–0·43)	<0·0001	..

Data for time since last event were only available in 12 839 patients.
