# Supplementary material for: Effects of aspirin on risk and severity of early recurrent stroke after transient ischaemic attack and ischaemic stroke: time-course analysis of randomised trials
Source: Lancet. 2016 Jul 23;388(10042):365–75. doi: 10.1016/S0140-6736(16)30468-8 (PMC5321490; doi:10.1016/S0140-6736(16)30468-8)
Supplement: Supplementary appendix [file mmc1.pdf]

THE LANCET

Supplementary appendix

This appendix formed part of the original submission and has been peer reviewed. We post it as supplied by the authors.

Supplement to: Rothwell PM, Algra A, Chen Z, Diener H-C, Norrving B, Mehta Z. Effects of aspirin on risk and severity of early recurrent stroke after transient ischaemic attack and ischaemic stroke: time-course analysis of randomised trials. *Lancet* 2016; published online May 18. http://dx.doi.org/10.1016/S0140-6736(16)30468-8.

Webappendix: Pothwell PM, Algra A, Chen Z, Diener H-C, Norrving B, Mehta Z. Effects of aspirin on risk and severity of early recurrent stroke after transient ischaemic attack and ischaemic stroke: time-course analysis of randomised trials.

A population-based study (Oxford Vascular Study) of time-trends in patient behavior after TIA and minor stroke versus major stroke in relation to the FAST television campaigns in the UK. The shaded areas reflect the timing of the campaigns. Non-emergency presentation refers to patients making appointments to see their family doctor as opposed to presenting to the emergency department or calling for an ambulance. The lower graphs show the proportions of patients first seeking medical attention within 3 hours and within 24 hours.

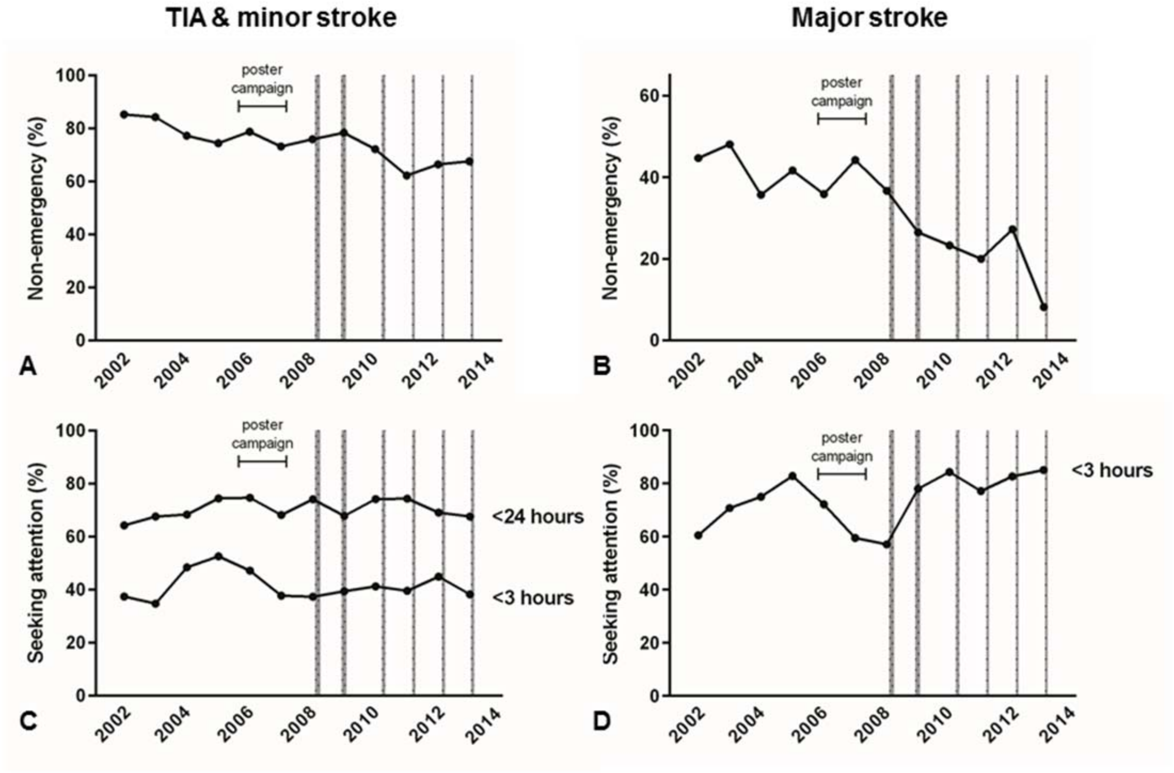

Courtesy of Dr Frank J. Wolters. Updated analysis from:

1. Wolters, FJ, Paul, NLM, Chandratheva, A et al. Contrasting impact of FAST-test public education campaign on behaviour after TIA and minor stroke versus major stroke: a population-based study. Cerebrovascular Diseases 2013, 35, 719-20.

2. Wolters FJ, Paul NL, Li L, Rothwell PM; Oxford Vascular Study. Sustained impact of UK FAST-test public education on response to stroke: a population-based time-series study. Int J Stroke 2015; 10:1108-14.

Severity of recurrent vascular events in the first 90-days after seeking medical attention in patients with TIA and minor stroke ascertained in the EXPRESS study in Phase-1 (standard treatment) versus Phase-2 (urgent treatment).[1,2] The effect of urgent treatment increased with increasing severity of recurrent event (interaction – p=0.002).

	**Phase-1**	**Phase-2**
Patients	310	281
Recurrent events		
TIA	17	15
Non-disabling stroke #	16	5
Disabling or fatal stroke #	16	1

Outcome is based on the modified Rankin score (mRS) at 1-3 months after the recurrent event: non-disabling stroke (mRS 0-2); Disabling or fatal stroke (mRS 3-6).

The 90-day risk of recurrent stroke after first seeking medical attention in all patients with TIA or stroke referred to the EXPRESS Study Clinic. The thick line represents phase-1 of the EXPRESS Study and the thin line represents phase-2.

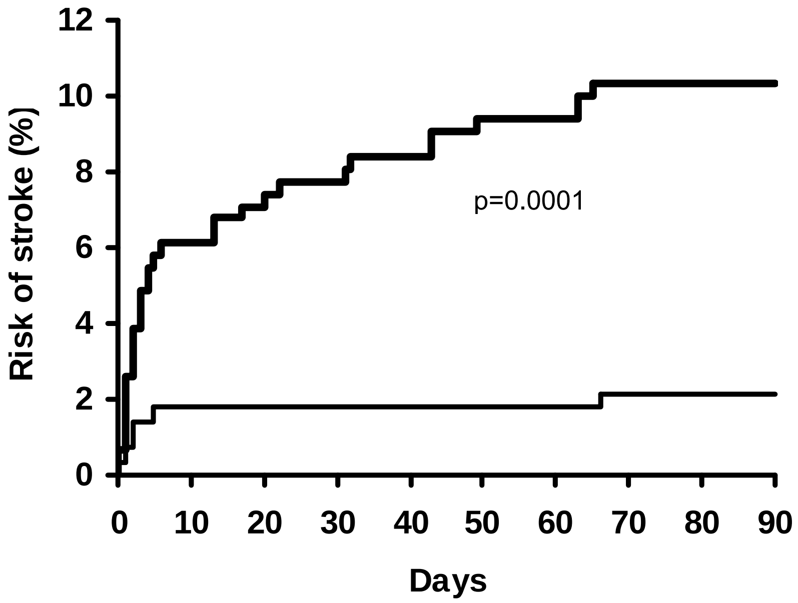

1. Rothwell PM, Giles MF, Chandratheva A, et al, on behalf of the Early use of Existing Preventive Strategies for Stroke (EXPRESS) Study. Major reduction in risk of early recurrent stroke by urgent treatment of TIA and minor stroke: EXPRESS Study. Lancet 2007; 370: 1432-42.

2. Luengo-Fernandez R, Gray AM, Rothwell PM. Effect of urgent treatment for transient ischaemic attack and minor stroke on disability and hospital costs (EXPRESS study): a prospective population-based sequential comparison. Lancet Neurol. 2009; 8:235-43.

Details of the trials of aspirin and/or dipyridamole in secondary prevention after TIA and ischaemic stroke included in analyses.

Comparison / Trial	Participants	Male (%)	Mean (SD) age (years)	Diabetes (%)	Hypertension (%)	Aspirin dose (mg)	Mean duration
Aspirin alone vs no antiplatelet drug							
AITIA [1]	319	70%	58 (14)	-	-	1200	17
UK-TIA [2]	2435	73%	60 (9)	4%	27%	300/1200	50
Reuther [3]	60	65%	58 (10)	17%	50%	1500	24
CA Co-op [4]	283	67%	61 (9)	8%	37%	1300	26
Toulouse TIA [5]	303	86%	63 (9)	-	-	900	34
AICLA [6]	402	68%	64 (10)	23%	64%	900	36
Danish Co-op [7]	203	73%	59 (9)	6%	-	1000	33
Swedish Coop [8]	505	62%	68 (10)	17%	46%	1500	24
Danish Low Dose [9]	301	65%	59 (8)	7%	-	50	23
SALT [10]	1359	66%	67 (7)	13%	47%	75	32
ESPS-2 [11,12]	3298	58%	67 (11)	15%	61%	50	24
Aspirin + dipyridamole vs no antiplatelet drug							
ESPS-1 [13]	2500	58%	-	-	-	975	23
Toulouse TIA [5]	293	86%	63 (9)	-	-	900	34
AICLA [6]	406	68%	64 (10)	23%	64%	900	36
ESPS-2 [11,12]	3299	58%	67 (11)	15%	61%	50	24
Aspirin + dipyridamole vs aspirin							
ACCSG [14]	890	67%	63	15%	48%	1300	25
ESPS-2 [11,12]	3299	58%	67 (11)	15%	61%	50	24
ESPRIT [15]	2763	66%	63 (11)	19%	60%	30-325	42
EARLY [16]	548	62%	68	24%	74%	50	7 days
JASAP [17]	1295	72%	66 (8)	40%	88%	50/81	15
Toulouse TIA [5]	284	86%	63 (9)	-	-	900	34
AICLA [6]	400	68%	64 (10)	23%	64%	900	36
Dipyridamole vs no antiplatelet drug							
ESPS-2 [11,12]	3303	58%	67 (11)	15%	61%	50	24

References

1. Fields WS, Lemak NA, Frankowski RF, Hardy RJ. Controlled trial of aspirin in cerebral ischemia. Stroke 1977;8:301-14

2. Farrell B, Godwin J, Richards S, Warlow C. The United Kingdom transient ischaemic attack (UK-TIA) aspirin trial: final results. J Neurol Neurosurg Psychiatry 1991;54:1044-1054

3. Reuther R, Dorndorf W, Loew D. The treatment of transitory ischemic attacks with acetylsalicylic acid: results of a double-blind-study. MMW Munch Med Wochenschr 1980; 122: 795-8.

4. Canadian Co-operative Study Group. A randomised trial of aspirin and sulfinpyrazone in threatened stroke. N Engl J Med 1978;299:53-9

5. Toulouse TIA. Guiraud-Chaumeil B, Rascol A, David J, Boneu B, Clanet M, Bierme R. Prévention des récidives des accidents vasculaires cérébraux ischémiques par les anti-agrégants plaquettaires. Rev Neurol (Paris) 1982;138:367-85

6. Bousser MG, Eschwège E, Haguenau M, Lefauconnier JM, Thibult N, Touboul D, et al. "AICLA" controlled trial of aspirin and dipyridamole in the secondary prevention of athero-thrombotic cerebral ischaemia. Stroke 1983;14:5-14.

7. Sorensen PS, Pedersen H, Marquardsen J, Petersson H, Heltberg A, Simonsen N, et al. Acetylsalicylic acid in the prevention of stroke in patients with reversible cerebral ischemic attacks. A Danish cooperative study. Stroke 1983;14:15-22.

8. Britton M, Helmers C, Samuelsson K. High-dose acetylsalicylic acid after cerebral infarction. A Swedish co-operative study. Stroke 1987;18:325-34.

9. Boysen G, Soelberg-Sørensen P, Juhler M, Andersen AR, Boas J, Olsen JS, et al. Danish very-low-dose aspirin after carotid endarterectomy trial. Stroke 1988;19:1211-5.

10. SALT Collaborative Group. Swedish aspirin low-dose trial (SALT) of 75mg aspirin as secondary prophylaxis after cerebrovascular ischaemic events. Lancet 1991;338:1345-9

11. Diener HC, Cunha L, Forbes C, Sivenius J, Smets P, Lowenthal A. European Stroke Prevention Study 2. Dipyridamole and acetylsalicylic acid in the secondary prevention of stroke. J Neurol Sci 1996;143:1-13.

12. ESPS 2 Group. European stroke prevention study: 2. Efficacy and safety data. J Neurol Sci 1997; 151(suppl): S1-77

11. Gent M, Barnett HJM, Sackett DL, Taylor DW. A randomized trial of aspirin and sulfinpyrazone in patients with threatened stroke. Results and methodologic issues. Circulation 1980;62(suppl V):97-105

13. The ESPS Group. The European Stroke Prevention Study (ESPS). Principal end-points. Lancet 1987; 2:1351-4.

14. The American-Canadian Co-Operative Study group. Persantine Aspirin Trial in cerebral ischemia. Part II: Endpoint results. Stroke 1985; 16: 406-15

15. The ESPRIT Study Group. Aspirin plus dipyridamole versus aspirin alone after cerebral ischaemia of arterial origin (ESPRIT): a randomised controlled trial. Lancet 2006;367:1665-73.

16. Dengler R, Diener HC, Schwartz A, Grond M, Schumacher H, Machnig T, Eschenfelder CC, Leonard J, Weissenborn K, Kastrup A, Haberl R; EARLY Investigators.Early treatment with aspirin plus extended-release dipyridamole for transient ischaemic attack or ischaemic stroke within 24 h of symptom onset (EARLY trial): a randomised, open-label, blinded-endpoint trial. Lancet Neurol 2010; 9: 159-66.

17. Uchiyama S, Ikeda Y, Urano Y, Horie Y, Yamaguchi T. The Japanese aggrenox (extended-release dipyridamole plus aspirin) stroke prevention versus aspirin programme (JASAP) study: a randomized, double-blind, controlled trial. Cerebrovasc Dis 2011; 31: 601-13.

Meta-analyses of the effects of aspirin (any aspirin vs control and aspirin only vs control) on risk of any recurrent ischaemic stroke and any disabling ischaemic stroke within 12 weeks of randomisation.

	**Ischaemic Stroke**			
	Any Aspirin	Control	**OR**	**95% CI**
UKTIA	25 / 1631	25 / 817	0.49	0.28-0.86
SALT	9 / 676	21 / 683	0.43	0.19-0.94
ESPS1	16 / 1250	30 / 1250	0.53	0.29-0.97
ESPS2	61 / 3299	132 / 3303	0.45	0.33-0.62
8 small trials	21 / 1596	39 / 1273	0.42	0.25-0.72
**TOTAL**	132 / 8452	247 / 7326	0.46	0.37-0.57

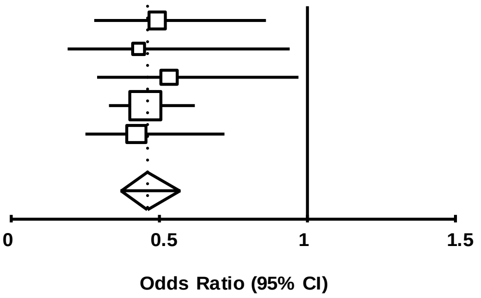

Significance: p <0.0001
Heterogeneity: p = 0.98

	**Ischaemic Stroke**			
	Aspirin only	Control	**OR**	**95% CI**
UKTIA	25 / 1631	25 / 817	0.49	0.28-0.86
SALT	9 / 676	21 / 683	0.43	0.19-0.94
ESPS2	31 / 1649	75 / 1649	0.40	0.26-0.61
8 small trials	21 / 1257	39 / 1273	0.54	0.31-0.92
**TOTAL**	86 / 5213	160 / 4422	0.46	0.35-0.60

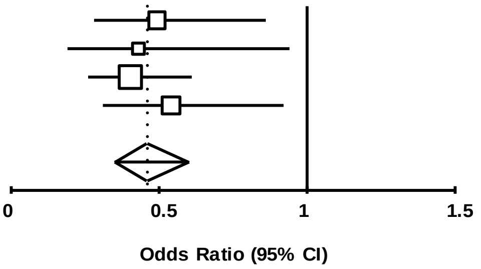

Significance: p <0.0001
Heterogeneity: p = 0.85

	**Disabling Ischaemic Stroke**			
	Any Aspirin	Control	**OR**	**95% CI**
UKTIA	10 / 1631	17 / 817	0.29	0.13-0.64
SALT	3 / 676	8 / 683	0.38	0.10-1.42
ESPS1	3 / 1250	8 / 1250	0.37	0.10-1.41
ESPS2	34 / 3299	95 / 3303	0.35	0.24-0.52
8 small trials	9 / 1596	23 / 1273	0.31	0.14-0.67
**TOTAL**	59 / 8452	151 / 7326	0.34	0.25-0.46

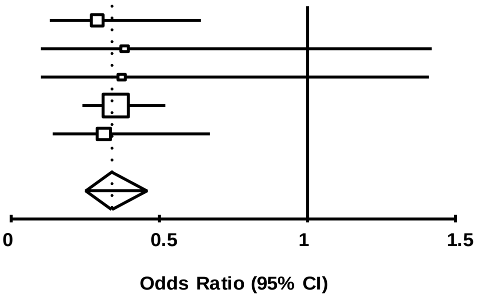

Significance: p <0.0001
Heterogeneity: p = 0.99

	**Disabling Ischaemic Stroke**			
	Aspirin only	Control	**OR**	**95% CI**
UKTIA	10 / 1631	17 / 817	0.29	0.13-0.64
SALT	3 / 676	8 / 683	0.38	0.10-1.42
ESPS2	19 / 1649	55 / 1649	0.34	0.20-0.57
8 small trials	9 / 1257	23 / 1273	0.39	0.18-0.85
**TOTAL**	41 / 5213	103 / 4422	0.34	0.24-0.49

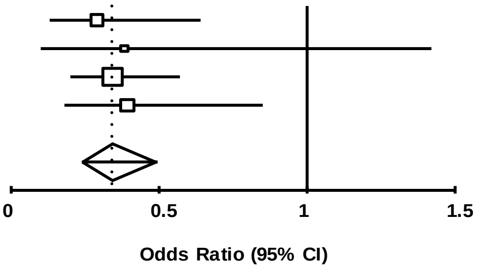

Significance: p <0.0001
Heterogeneity: p = 0.96

Pooled analysis of the effect of any aspirin vs control in secondary prevention after TIA and ischaemic stroke on the early risk of any recurrent ischaemic stroke and on disabling or fatal ischaemic stroke stratified by baseline clinical characteristics. Complete baseline data available on only the 10,409 participants in UKTIA, SALT and ESPS-2.

	HR	LL	UL	p	p(interaction)
**All ischaemic stroke**					
**Age**					0.95
<65	0.50	0.33	0.74	0.0006	
65-74	0.39	0.26	0.57	<0.0001	
≥75	0.54	0.36	0.83	0.0049	
**Sex**					0.84
Female	0.45	0.30	0.67	<0.0001	
Male	0.48	0.36	0.64	<0.0001	
**Hypertension**					0.12
No	0.66	0.42	1.05	0.079	
Yes	0.40	0.30	0.53	<0.0001	
**Current smoking**					0.57
No	0.49	0.35	0.68	<0.0001	
Yes	0.42	0.28	0.63	<0.0001	
**Diabetes**					0.92
No	0.47	0.35	0.62	<0.0001	
Yes	0.43	0.24	0.77	0.0045	
**Aspirin Dose**					
Low (≤100mg)	0.51	0.37	0.71	<0.0001	0.53
High (≥300mg)	0.44	0.33	0.59	<0.0001	

Disabling or fatal ischaemic stroke

	HR	LL	UL	p	p(interaction)
**Age**					0.44
<65	0.33	0.17	0.66	0.0017	
65-74	0.27	0.16	0.45	<0.0001	
≥75	0.48	0.29	0.81	0.0056	
**Sex**					0.84
Female	0.34	0.20	0.57	<0.0001	
Male	0.36	0.24	0.54	<0.0001	
**Hypertension**					0.20
No	0.52	0.29	0.95	0.033	
Yes	0.29	0.19	0.43	<0.0001	
**Current smoking**					0.45
No	0.39	0.25	0.60	<0.0001	
Yes	0.30	0.18	0.51	<0.0001	
**Diabetes**					0.77
No	0.36	0.25	0.52	<0.0001	
Yes	0.30	0.14	0.67	0.0034	
**Aspirin dose**					
Low (≤100mg)	0.33	0.20	0.55	<0.0001	0.82
High (≥300mg)	0.36	0.24	0.52	<0.0001	

Meta-analysis of the effect aspirin only vs. control on the risk of recurrent ischaemic stroke more than 12 weeks after randomisation in trials of aspirin in secondary prevention after TIA and ischaemic stroke.

This preliminary analysis includes strokes known to be ischaemic and strokes of unknown type and the UK-TIA trial outcomes are confined to the 'major stroke; primary outcome (i.e. events with symptoms lasting longer than 7 days). The denominators are the numbers of patients at randomisation rather than at 12 weeks. The analysis is by intention-to-treat and so no account is taken of compliance with randomised treatment allocation. More detailed analyses are ongoing.

	**Ischaemic Stroke**		**OR**	**95% CI**
	Aspirin only	Control		
UKTIA	130 / 1631	67 / 817	0.97	0.71-1.32
SALT	64 / 676	71 / 683	0.90	0.63-1.29
ESPS2	157 / 1649	150 / 1649	1.05	0.83-1.33
8 small trials	94 / 1257	105 / 1273	0.90	0.67-1.20
**TOTAL**	445 / 5213	393 / 4422	0.97	0.84-1.12

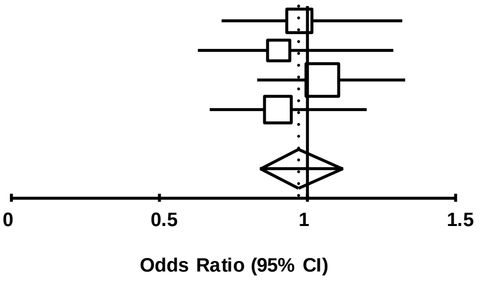

Significance: p = 0.67

Heterogeneity: p = 0.83

Details of the trials aspirin vs control in treatment of acute ischaemic stroke that were included in analyses.

CAST [1]
Double blind
21,106 participants
63% male
28% were more than 70 years old
87% had CT before entry
Ischaemic stroke less than 48 hours since stroke onset
Interventions Rx: aspirin 160 mg once daily orally or via nasogastric tube
Control: placebo
Duration: 4 weeks, or until death or earlier discharge
FU: 4 weeks

IST [2]
Unblinded
19,435 participants
54% male
61% more than 70 years
67% CT prior to randomisation, 29% CT after randomisation
Ischaemic stroke less than 48 hours since stroke onset
Interventions Rx: subcutaneous heparin (5000 IU or 12 500 IU 12 hourly), aspirin 300 mg, both, or neither (factorial design)
Duration: 14 days or until discharge from hospital
Interventions Rx: Aspirin by mouth if able to swallow, if not then by rectal suppository or by injection of 100 mg of the lysine salt of aspirin
FU: 6 months

Rödén-Jüllig [3]
Double blind
441 participants
226 (51%) male
100% CT before entry
Ischaemic stroke less than 72 hours since stroke onset
Interventions Rx: aspirin 325 mg orally once daily
Control: placebo
Duration: 5 days
FU: 3 months

References

1. CAST (Chinese Acute Stroke Trial) Collaborative Group. CAST: randomised placebo-controlled trial of early aspirin use in 20,000 patients with acute ischaemic stroke. Lancet 1997; 349: 1641-9.

2. International Stroke Trial Collaborative Group. The International Stroke Trial (IST): a randomised trial of aspirin, subcutaneous heparin, both, or neither among 19435 patients with acute ischaemic stroke. Lancet. 1997; 349: 1569-81.

3. Rödén-Jüllig A, Britton M, Malmkvist K, Leijd B. Aspirin in the prevention of progressing stroke: a randomized controlled study. J Intern Med 2003; 254: 584-90.

Meta-analysis of the effect of aspirin vs control on the 14-day risk of recurrent ischaemic stroke in trials of aspirin in treatment of acute stroke stratified by the extent of the initial neurological (mild, moderate or severe).

Heterogeneity between 3 severity groups: p=0.014

	Events / Patients		OR	95% CI
	Aspirin	Placebo		
**Mild**				
IST	24 / 2025	44 / 2026	0.54	0.33-0.89
CAST	10 / 2182	24 / 2231	0.42	0.20-0.89
Roden-Jullig	4 / 47	7 / 50	0.57	0.16-2.10
**TOTAL**	38 / 4254	75 / 4307	0.51	0.34-0.75

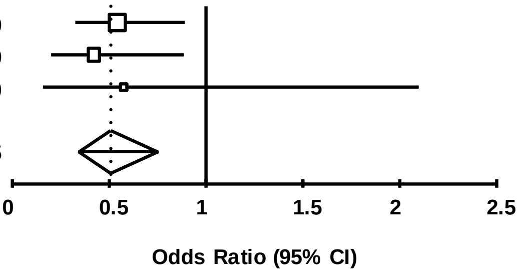

Significance: p = 0.0007
Heterogeneity: p = 0.85

	Events / Patients		OR	95% CI
**Moderate**				
IST	61 / 4498	127 / 4455	0.47	0.34-0.64
CAST	88 / 7256	113 / 7171	0.77	0.58-1.02
Roden-Jullig	16 / 108	19 / 120	0.92	0.45-1.90
**TOTAL**	165 / 11862	259 / 11746	0.65	0.44-0.98

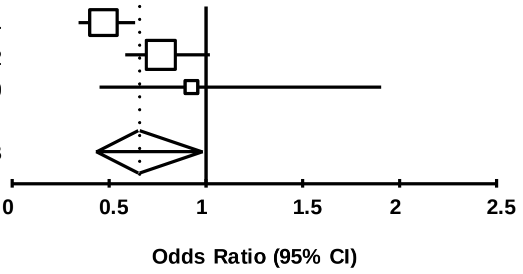

Significance: p = 0.040
Heterogeneity: p = 0.039

	Events / Patients		OR	95% CI
**Severe**				
IST	30 / 1236	22 / 1255	1.39	0.80-2.43
CAST	23 / 890	27 / 910	0.87	0.49-1.52
Roden-Jullig	15 / 65	11 / 51	1.09	0.45-2.64
**TOTAL**	68 / 2191	60 / 2216	1.10	0.77-1.58

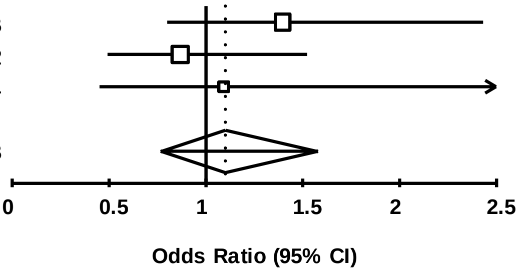

Significance: p = 0.60
Heterogeneity: p = 0.50

Pooled hazard ratios for the effect of aspirin vs control on risk of recurrent ischaemic stroke in patients with mild and moderately severe initial neurological deficits during early follow-up (days 0-1; 2-3,4-6; 7-14; >15) in two large trials of aspirin in treatment of acute stroke. This analysis is equivalent to figure 3 in the main body of the paper except that it includes 3292 (21.3%) patients with mild or moderately severe stroke in the International Stroke trial who had received aspirin during the days prior to randomisation.

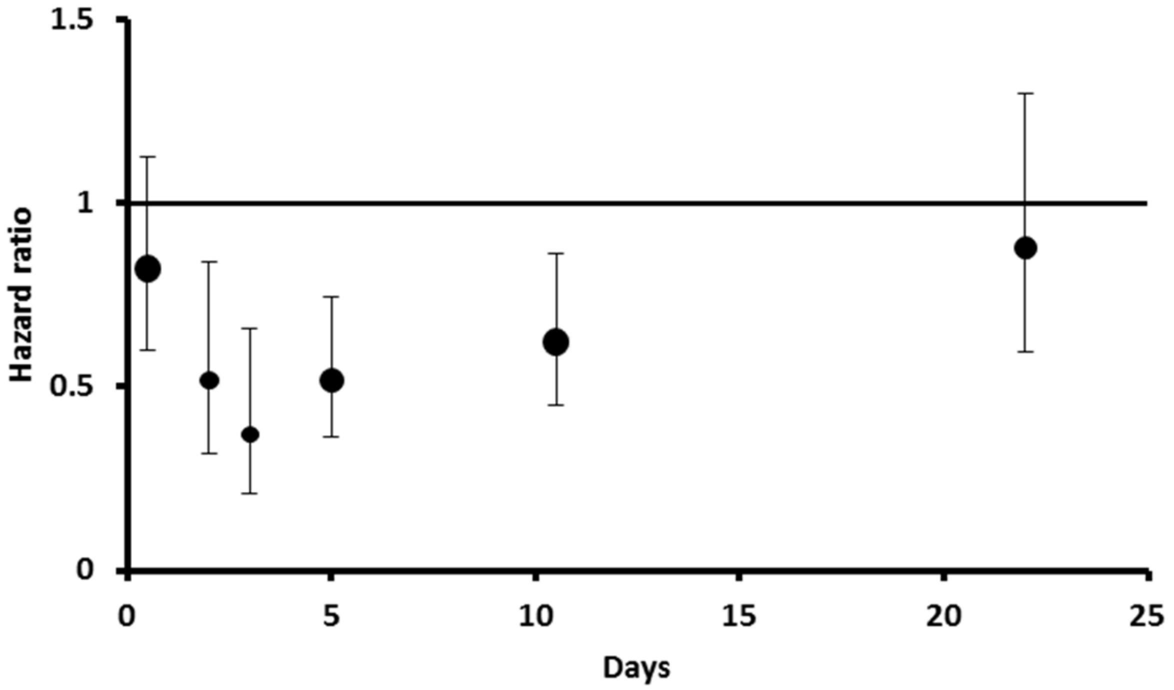

Meta-analysis of the effect dipyidamole plus aspirin versus aspirin alone on the risk of recurrent ischaemic stroke within 12 weeks after randomisation in trials in secondary prevention after TIA and ischaemic stroke.

	**Ischaemic Stroke**		**OR**	**95% CI**
	Aspirin+ Dipyridamole	Aspirin		
EARLY	9 / 278	13 / 270	0.66	0.28-1.57
JASAP	14 / 652	6 / 639	2.32	0.88-6.06
ACCSG	8 / 448	12 / 442	0.65	0.26-1.61
ESPRIT	11 / 1353	15 / 1371	0.74	0.34-1.62
ESPS2	30 / 1650	31 / 1649	0.97	0.58-1.60
2 small trials	0 / 340	2 / 345	0.20	0.01-4.22
**TOTAL**	72 / 4721	79 / 4716	0.90	0.65-1.25

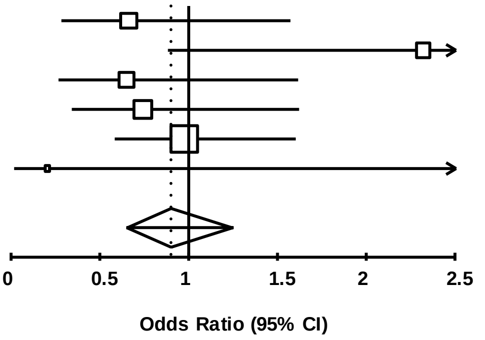

Significance: p = 0.53
Heterogeneity: p = 0.31
